# P40 A challenging case of new systemic lupus erythematosus

**DOI:** 10.1093/rap/rkae117.071

**Published:** 2024-11-05

**Authors:** Hassan Shah, Rainer Klocke, Sunil Nadar

**Affiliations:** Dudley Group NHS Trust, Dudley, United Kingdom; Dudley Group NHS Trust, Dudley, United Kingdom; Dudley Group NHS Trust, Dudley, United Kingdom

## Abstract

**Introduction:**

The diagnosis of systemic lupus erythematosus (SLE) and its distinction from systemic infection can be challenging. We present a case of new systemic lupus alongside endocarditis, which illustrates this problem.

**Case description:**

A previously well, 35-year-old male tarmacer of mixed ethnicity, presented with a three months’ history of general malaise, arthralgia and weight loss of 20kg. Two weeks prior to admission he developed progressive confusion, headaches and imbalance. Clinical assessment revealed a pyrexial patient in delirium without focal neurological signs, a faint maculopapular facial rash, symmetrical synovitis, very few nailfold infarcts, a systolic and diastolic murmur and sterile haemato-proteinuria. No history of IV drug abuse.

Investigations showed elevated ESR 61, CRP 217, WBC 11.70 and procalcitonin 7.0. He had AKI stage 2 (eGFR 35 with a urinary protein/creat ratio 162; homogenous ANA (1:1280) with positive dsDNA and crithidia-luciliae binding. C3 and C4 suppressed (0.59 and 0.13, respectively);

One blood culture prior to broad spectrum antibiotics was negative. Assuming the possibility of Libman-Sacks endocarditis, the attending acute stroke physician initially also treated with pulsed IV steroids, as well as antibiotics. Due to the delirium, key diagnostic tests were technically difficult, although a transthoracic ECHO showed moderately severe aortic and mitral regurgitation that were not felt to be typical for infective endocarditis by the attending cardiologist.

The ITU team offered sedation and controlled ventilation to enable all necessary investigations: trans-oesophageal ECHO showed vegetations of aortic and mitral valve leaflets with perforations of single leaflets, resulting in severe acute aortic and mitral regurgitation; a renal biopsy showed class 3 lupus nephritis and an MRI/A Brain showed widespread cerebral foci of restricted diffusion imaging.

Consultation with the regional cardiothoracic centre advised urgent transfer for double metal valve replacement, performed on Day 8 post admission. Surgical inspection confirmed an aortic root abscess, and enrichment culture of the native valve subsequently grew Staph. aureus.

In addition to IV antibiotics for infective endocarditis, he was continued on steroid taper and commenced on MMF for lupus nephritis.

**Discussion:**

It can be difficult to differentiate between infective endocarditis and Libman-Sacks endocarditis, especially when there are no existing risk factors. In cases like these antibiotics and steroids can be started at the same time.

In our case, we had a young gentleman with no prior medical history who had multiple compounding factors and barriers to fully investigate.

Because of his agitation, he required sedation and that allowed the team to perform life saving investigations (renal biopsy and TOE). He ended up having a double metal valve replacement within 8 days post-admission.

Multidisciplinary team involvement and close collaboration was of crucial importance in this case to ensure a safe patient outcome.

**Key learning points:**

• This case illustrates the need to vigorously chase the possibility of concurrent infection in severe new SLE. Close collaboration between multiple specialities to achieve this is key, especially when a patient lacks agency due to their illness.

